# Exploring the acceptance of virtual reality training systems among construction workers: a combined structural equation modeling and artificial neural network approach

**DOI:** 10.3389/fpubh.2024.1478615

**Published:** 2024-12-18

**Authors:** Hui Liu, Xinyue Miao, Chunyan Shi, Tongyu Xu

**Affiliations:** ^1^School of Emergency Science and Engineering, Jilin Jianzhu University, Changchun, China; ^2^The University of Kitakyushu, Fukuoka, Japan

**Keywords:** virtual reality training system, construction workers, technology acceptance model, theory of planned behavior, artificial neural network

## Abstract

Virtual Reality Training System (VRTS) has been verified effective in safety training in the construction field. However, in China, it is not widely used as a regular training tool. Among all the reasons, the acceptance level of construction workers (CWs) has the decisive impact on the promotion of VRTS. In view of this, this study is devoted to constructing a training model of CWs’ acceptance level of VRTS training that integrates the technology acceptance level model with the theory of planned behavior. What’s more, this paper innovatively introduces three crucial elements of external influences, namely, risk perception (RP), safety climate (SC) and self-efficacy (SE). In order to more accurately figure out the linear and nonlinear relationship between every structure and the factors of CWs’ acceptance level, 528 participating CWs in this study filled in structured questionnaires, through the data of which the analyzing process uses structural equation model and artificial neural network two-stage analysis method. Based on the analyzing results of the study, this paper put forward a series of specific strategies and suggestions to significantly promote the acceptance level of CWs to VRTS training considering the designment, the enterprises and the government.

## 1 Introduction

The construction industry consistently exhibits high rates of work-related injuries and fatalities, surpassing other sectors globally (1). Construction workers (CWs) face significantly higher risks with a threefold increased likelihood of fatality and twice the likelihood of sustaining injuries compared to workers in other industries (2). It all boils down to unsafe CWs’ behaviors. In view of this, CWs’ education and training is needed if reducing accidents in the construction field. Safety training has been proven to be crucial in enhancing safety awareness among CWs and effectively mitigating their unsafe behaviors, thereby preventing potential accidents at construction sites (3). Historically, various methods have been employed to provide safety training for CWs, including video recordings, hand-outs, and hands-on training. However, these training methods require CWs to pay special attention to the effectiveness of learning during training, otherwise it will fail to change the safety objectives of the construction sites (4).

Research has shown that as the attractiveness of the training methodology increases, CWs demonstrate higher levels of knowledge acquisition, while the incidence of workplace accidents decreases (5). The advent of VR training has been shown to be more effective in engaging the attention of people (6), and it is successfully used in many scientific fields, such as in the military (7), education, mining, medical therapy and sports. In the field of construction (8), the outstanding advantage of VR training is to visualize complex construction environment, enabling CWs to actively explore this virtual environment (9). It not only enhances their immersion, but also provide them with a deeper understanding and appreciation of safety training. The application of VR training in simulating the consequences of accidents demonstrates real-time and realistic visual effects. Immersive platforms are effective in visualizing and identifying hazards, which helps in developing hazard avoidance strategies (10, 11). Examples include the simulation of falling scenarios in working at heights, object strikes in floor collapses, electrocution scenarios under the chaotic placement of electrical wiring, etc. The simulation of these scenarios in VR enables multiple repetitions of the operation and ensures that CWs are not affected by potential hazards in order to gain a more comprehensive understanding of the various safety risks in the construction field, to improve safety awareness, and to master countermeasures. Therefore, the adoption of VR training is seen as a key means to effectively reduce unsafe behaviors of CWs and improve the quality of works.

Extensive research has consistently showcased the superior effectiveness of VRTS in comparison to conventional training methods. Nonetheless, despite the remarkable advantages of VRTS, their introduction as a novel safety training method in the construction field has not been widely adopted as a conventional approach to safety training (12). The main reason for this phenomenon is because CWs, as the subject of learning and using VRTS, are not clear in their acceptance degree and behavioral intention to facilitate the effective use of VRTS in construction safety training. In this context, three questions have been raised:

1. How is the acceptance level of CWs to VRTS at the recent stage in China?
2. What are the factors that affect CWs’ attitudes toward and intention to use VRTS?
3. Can we consider using VRTS as an alternative to traditional training methods in China? At the same time, how can we effectively increase CWs’ acceptance level through a VRTS-based training model and further promote the widespread use of VRTS in China?

The empirical validation of the TAM has established its efficacy as a valuable theoretical framework for understanding users’ attitudes toward adopting new technologies, encompassing behavioral attitudes related to PU and PEU, as well as users’ intention to directly or indirectly adopt these technologies (13, 14). The validation is supported by extensive empirical evidence. However, despite TAM is a minimalistic and robust model, it utilizes only two variables to explain behavioral intention. Behavioral intention is also influenced by other factors, including the opinions of significant individuals (subjective norms) (15) and the availability of resources and skills (perceived behavioral control) (16). A new model, which is named TPB, drives behavioral intention and influences behaviors through ATT, subjective norms (SN) and perceived behavioral control (PBC) (17).

This paper innovatively extends TAM and, for the first time, combines it with TPB to create a theoretical framework to illustrate the acceptance of VRTS by CWs. In recent years, in the study of VRTS’ acceptance by CWs, many researchers discuss the influence of perceived playfulness (12), age, past use (18) and some other influence behavioral intentions of CWs. In addition, studies have proved the significance of RP and SC in elucidating the factors contributing to unsafe behaviors among CWs (19), which is also used in the study of usage intention of CWs (20). To provide a comprehensive understanding of the acceptance of VRTS among CWs, a model is developed that incorporates three key factors: SC, RP, and SE. Additionally, for the first time, attitudes are divided into hardware attitude and content attitude, combining the TAM and TPB. Through thorough analysis, this study provides valuable insights and offers corresponding policy recommendations from multiple perspectives. The findings of this research reflect the current use and acceptance of VRTS by CWs in Shenzhen, China, and serve as a foundational basis for assessing the feasibility of promoting VRTS on a broader scale across China. The result of the study evaluates their acceptance level based on the attitude and willingness from CWs in Shenzhen, China. Besides, forementioned result will be the fundamental principle of promoting VRTS.

Initially, Steuer defined VR without reference to any hardware as ‘the experience of a perceiver of a remotely presented real or simulated environment’ (21). Chen et al. further named this VR, which did not require the support of any tracking device (and did not rely on the use of hardware), desktop-based VR, which could be viewed online, mostly through a computer or handheld device. Online, however, does not provide the most immersive experience (22). Therefore, there is a need to explore hardware to enhance the sense of presence for examining VR content, which is reflected in the immersion and human-computer interaction experience (23–25). And Laycock et al. argued that haptic devices that provide force feedback through physical manipulators can provide a better understanding of how objects in virtual environments physically interact (26). Therefore, some researchers have argued that there is a need to use some necessary devices, hardware or objects to enhance the immersion and human-computer interaction experience during the VR experience. For example, the use of handheld controllers to navigate and manipulate objects in the virtual world allows for haptic feedback and enhanced interactivity (27, 28). Berg and Vance provide an updated alternative definition of virtual reality, including hardware components, defined as Immersive Computing Technology (ICT), ‘a set of technologies that enable immersive experiences of worlds beyond reality’. VR hardware is also defined as the device that allows users to interact, view and experience VR content (18). Kerry T. Manis argued that distinguishing between VR content and VR hardware is necessary, but that these terms could still be blended into a single synonym—virtual reality. In light of this, this study will delve into the effects of CWs on willingness to use VRTS along two dimensions: attitudes toward learning VR content and attitudes toward using VR hardware, respectively.

The structure of the paper is as follows. Section 2 introduces the theoretical framework and research hypotheses. Section 3 describes the research methodology and data analysis. Section 4 presents the empirical analysis results and discusses the main findings of the study. Section 5 presents discussion and policy implications, Section 6 provides conclusion and Section 7 summarizing the limitations of this research and offering suggestions for further studies.

## 2 Theoretical framework and research hypothesis

### 2.1 Technology acceptance model

**Figure 1:**
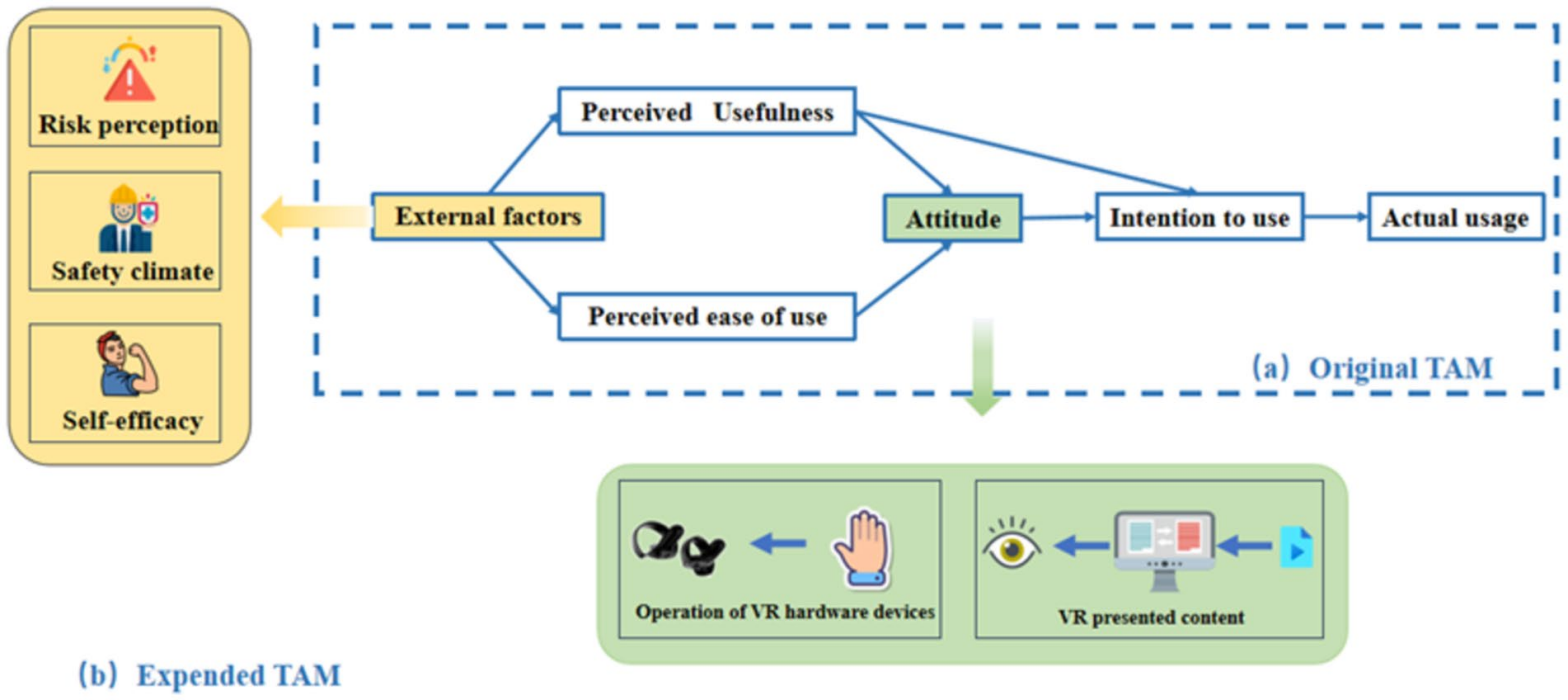
Framework diagram of TAM original model to extended model.

Davis (29) derived the TAM from the Theory of Reasoned Action (TRA) as its foundation (15), and found that it could better explain users’ acceptance of a certain technology (29). Figure 1A shows that the TAM. Perceived Ease of Use (PEU), Perceived Usefulness (PU), and Attitudes (ATT) toward technology usage are the key factors that explain individuals’ intentions to use it. An individual’ s actual behavior is determined by behavioral intention, and the intention is determined by both PU and ATT. ATT is influenced by PU and PEU, with PU being affected by both PEU and external variables, while PEU is solely influenced by external variables. TAM has been widely used in user acceptance studies of various technologies. For instance, Yaobin Lu discussed the Chinese users’ acceptance of instant messaging (30); Kerry T extended and personalized VR hardware’s TAM (18); Tom Ka Man Wong used TAM to explore the acceptance of the personal protective equipment by CWs (31); TAM was stable in all countries (32); and Ming Zhang employed an extended TAM to explore the reasons for the limited adoption of VR technology (12). Therefore, it could be concluded that the TAM is universal, and this study has improved the current model with the aim of exploring the acceptance level of VRTS among CWs. The research innovatively differentiates the attitudes of construction workers toward virtual reality hardware and virtual reality content, and deeply analyzes the impact of PU and PEU on these two different attitudes, as well as their roles in influencing behavioral intentions. Furthermore, considering that the working environment and personal ability to use VRTS may affect their acceptance, this study introduces three external variables based on existing literature: Role Perception (RP), Self-Efficacy (SE), and Situational Control (SC), as show in Figure 1B.

First, TAM uses two major internal beliefs (PU and PEU) to determine user attitudes toward a particular technology system (33). Davis (29) defined PU as ‘the degree to which people believe that using a particular system will improve his or her work performance’ and PEU as ‘the degree to which people believe that using a particular system is effortless’. In this study, PU is defined as the effect of CWs improving their own safety behavior through the use of VRTS. PEU is defined as the degree of how easy CWs consider it to use the VRTS. Secondly, in the TAM, ATT refers to a psychological construct that represents an individual’s evaluation, feelings, and predisposition toward a particular object, person, event, or concept. ATT reflects a person’s favorable or unfavorable disposition that can be positive, negative, or neutral (34, 35). ATT plays a crucial role in influencing an individual’s thoughts, emotions, and behaviors, and they can impact decision-making, interpersonal relationships, and overall perception of the world (36). According to the new perspective on VR described in the previous section, this paper defines ATT as the assessment and feelings of CWs when using VR hardware (ATT1) and the assessment and feelings of CWs when learning VR content (ATT2). Finally, in the TAM, the intention to use (IU) is defined as the degree of intention of users to use the system, and the paper defines it as the degree of willingness of CWs to use VRTS. In the TAM hypotheses, PU and PEU have a positive effect on users’ attitude; PEU has a positive effect on PU; PU and ATT has a positive effect on the IU. Therefore, the following hypotheses are proposed:

*H1*. Perceived ease of use has a positive effect on perceived usefulness.

*H2*. Perceived usefulness has a positive effect on attitude of VR content.

*H3*. Perceived usefulness has a positive effect on attitude of using VR hardware.

*H4*. Perceived usefulness has a positive effect on the intention to use.

*H5*. Perceived ease of use has a positive effect on attitude of VR content.

*H6*. Perceived ease of use has a positive effect on attitude of using VR hardware.

*H7*. Attitude of VR content has a positive effect on the intention to use.

*H8*. Attitude of using VR hardware has a positive effect on the intention to use.

### 2.2 Theory of planned behavior

**Figure 2:**
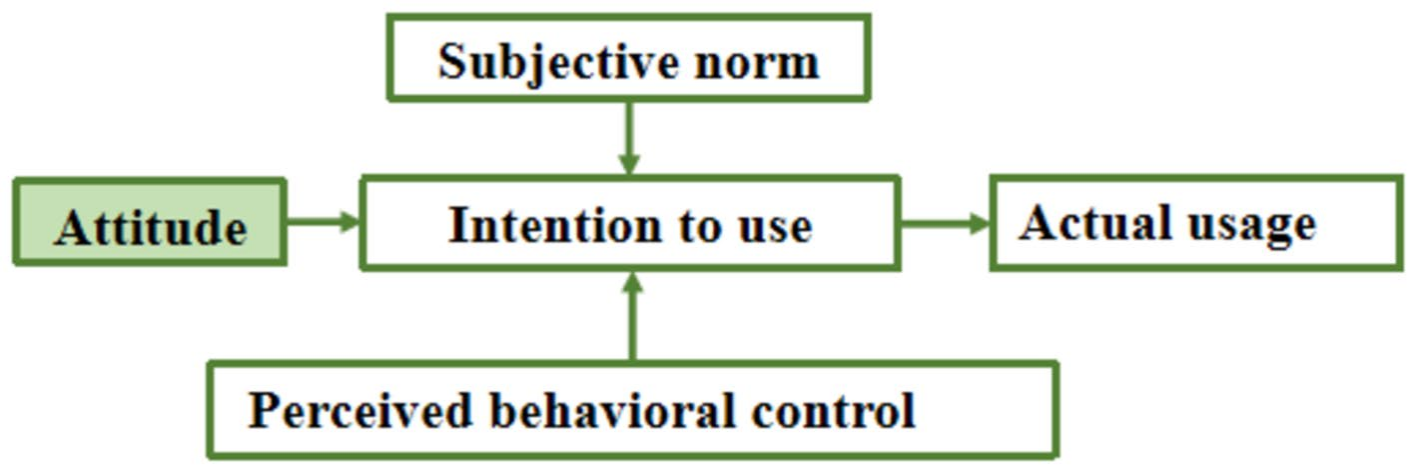
Framework diagram of TPB original model.

TAM can efficiently and accurately predict users’ intention to use a technology. However, its limitation is that it fails to comprehensively consider factors such as individual control and social cognition of new technologies. The TPB proposed by Ajzen effectively remedies the shortcomings of the TAM by introducing the two core elements of Subjective Norms (SN) and Perceived Behavioral Control (PBC) and provides insights into how these elements work together to influence an individual’s behavioral intentions (16). Fett et al. used the extended TPB to discuss and assess the main factors of acceptance of wireless EV charging (37); Ding Lili used TPB to investigate the acceptance of desalinated water utilization by residents (38); Caisheng Liao used Extended TPB to investigate the factors influencing urban residents’ intention to travel in a low-carbon manner (39). These studies provide valuable insights and empirical support for TPB in explaining the acceptance of VRTS by CWs. Figure 2 illustrates the extended TPB model applied to the study to gain a more comprehensive understanding of the motivations influencing CWs’ willingness to use, which can provide new insights into the development of strategies to advance the popularity of VRTS.

In the TPB, attitude toward the behavior, SN, and PBC, together to drive behavioral intention, which in turn affects the actual usage (16). SN refers to an individual’s perception of the social pressure or influence to conform the particular behavior. It represents the perceived social expectations, norms, and opinions of significant others, such as family, friends, peers, or society, regarding a specific behavior or action. SN captures the individual’s belief about whether individual would approve to engage in a particular behavior. PBC encompasses users’ perception of possessing the required resources, capabilities, and a sense of control to successfully engage in a particular behavior. While the TPB is a broad framework, this study has made modifications to better elucidate the acceptance of VRTS among CWs. Here, we define SN as the perception of CWs regarding the approval or endorsement of important individuals who they believe that want them to use VRTS. When CWs perceive that those significant individuals expect them to utilize VRTS, it increases the likelihood of their adoption. PBC is defined as the CWs’ perception of their individual capability to use VRTS. It is hypothesized that CWs with higher levels of behavioral control are more likely to use VRTS. The direct positive contribution of both subjective norms and perceived behavioral control to behavioral intentions has been widely validated in a number of domains (40, 41). Therefore, the following hypotheses are proposed:

*H9*. Subjective norm has a positive effect on intention to use.

*H10*. Perceived behavioral control has a positive effect on intention to use.

*H11*. Intention to use has a positive effect on actual usage.

### 2.3 Risk perception

Risk perception is an individual’s subjective assessment and judgment of potential risk (42). In research on the acceptance of VRTS by CWs, we define this as the perception of potential operational risk by CWs when they enter the field without using VRTS training. Risk perception (RP) has also been consistently shown to be an important factor in positively contributing to attitudes and willingness within numerous areas of technology acceptance research (43–45). For example, in Huijt’s exploration of sustainable energy technology acceptance, he suggests that RP has a positive effect on attitudes (46). Similarly, Siu Shing Man’s findings show that the severity of RP for CWs has a positive effect on the influence of PPE use attitude (20). Furthermore, Siu Shing Man also pointed out that RP is a key factor that influences the impact of drivers’ attitude and willingness to use self-driving cars (47). A psychometric tool called ‘CWs Risk Perception Scale’ assesses the risk perception scale (48). The scale demonstrates three dimensions of workers’ RP: RP-probability, RP-severity, and RP-worry and insecurity. These dimensions are negatively correlated with workers’ risk-taking behavior. In this study, we focus on the dimension of RP-worry and insecurity. And it is hypothesized that this perception may drive their wider adoption of VRTS, i.e., and enhance their attitude and willingness to use VRTS. Therefore, the following hypotheses are proposed:

*H12*. Risk perception has a positive effect on attitude of using VR content.

*H13*. Risk perception has a positive effect on attitude of using VR hardware.

*H14*. Risk perception has a positive effect on the intention to use.

### 2.4 Safety climate

To enhance safety performance, it is vital to investigate workplace factors that influence safety risk perception. This study introduces a predictive measure known as safety climate, which captures the safety response within construction enterprises. The safety management practices of construction enterprises can significantly impact CWs’ perceived usefulness of VRTS, as well as their perceptions of risk and attitudes (49, 50). Research studies have indicated that there is no direct association between SC and actual safety behavior (51, 52). Therefore, its relationship with certain mediating variables requires further exploration. SC independently influences safety risk perception. And workplaces characterized by a more positive safety atmosphere tend to exhibit higher levels of safety risk perception (53). Moreover, the safety environment plays a positive role in influencing CWs’ perceived usefulness of protective equipment (20). In a study, it was also observed that the safety environment positively influences the safety attitudes of employees in transportation companies (54). Therefore, the following hypotheses are proposed:

*H15*. Safety climate has a positive effect on perceived usefulness.

*H16*. Safety climate has a positive effect on attitude of using VR content.

*H17*. Safety climate has a positive effect on attitude of using VR hardware.

*H18*. Safety climate has a positive effect on risk perception.

### 2.5 Self-efficacy

SE pertains to individuals’ belief in their own capabilities to successfully perform specific tasks or achieve desired outcomes. It is not a measure of actual skills or knowledge but a subjective assessment of one’s own competence in a given domain. In this study, we define SE as the confidence of CWs’ speculations and judgments about their ability to complete the process of using VRTS. The strength of VRTS lies in user interaction with the VR environment, as previously mentioned. However, when CWs are exposed to VRTS, the following situations may arise: Firstly, the 3D effect of VRTS can lead to symptoms such as dizziness, fatigue, or nausea among CWs, thereby affecting their overall experience (55–57). Secondly, the complexity of VRTS can also impact the learning experience and satisfaction of CWs (25). Consequently, CWs may avoid using VRTS if they perceive themselves as incapable of utilizing such technologies. Hence, it is crucial to consider SE as an external variable. Those CWs with high SE are more likely to find it easy to use the VRTS, an assumption consistent with Mun Y. Yi, that users who possess a stronger sense of efficacy regarding the target system perceive it are more user-friendly (58). Therefore, the paper formulates the following hypotheses:

*H19*. Self-efficacy has a positive effect on perceived ease of use.

**Figure 3:**
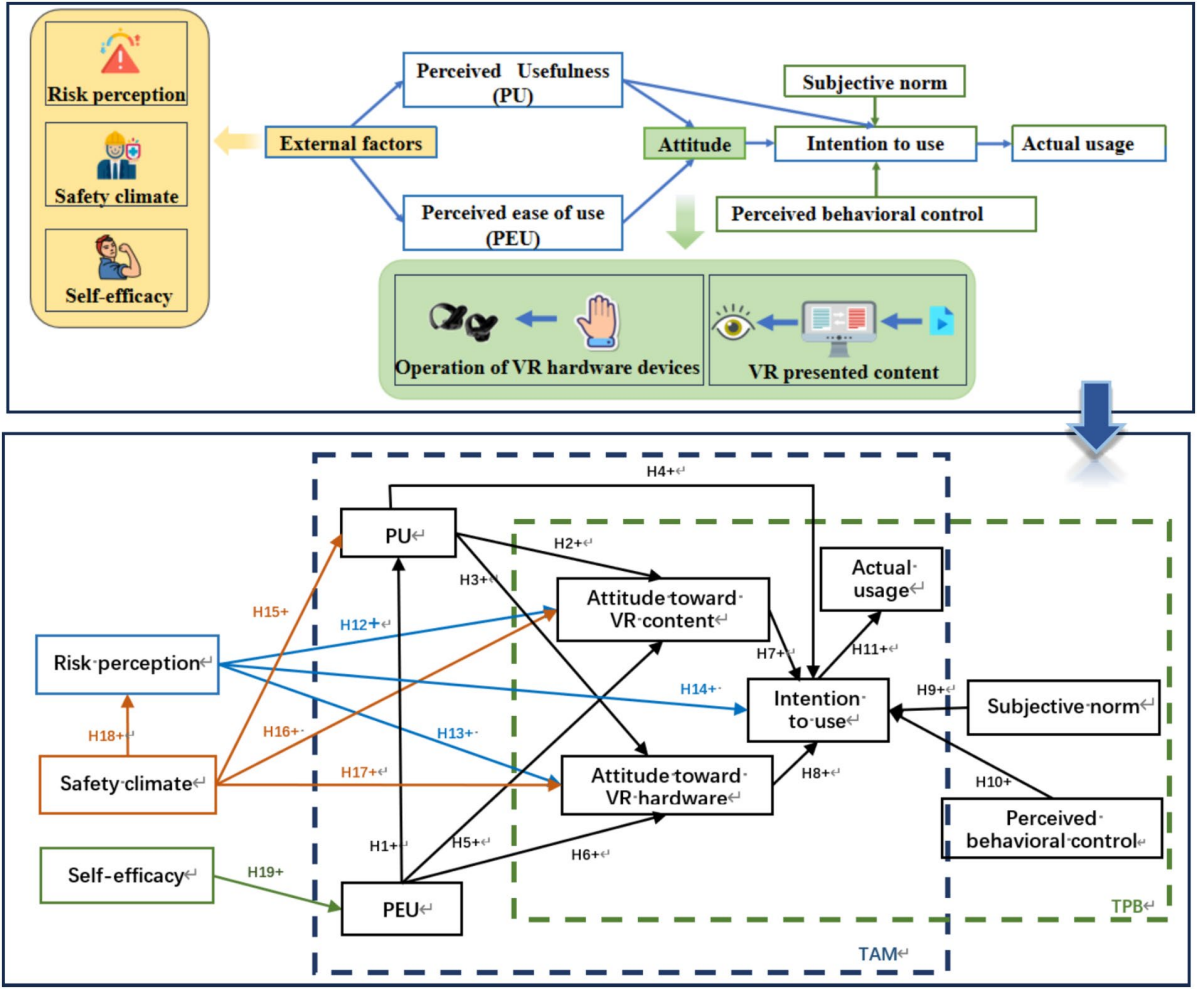
Theoretical framework of the extended TAM and TPB for VRTS usage.

Through the constructed hypothesis system, we recognize that in the TAM, perceived ease of use, perceived usefulness, and attitudes toward technology use are the key variables explaining an individual’s acceptance of technology. Specifically, an individual’s willingness to use a technology is driven by his or her perceived usefulness and attitude toward using the technology. This attitude, in turn, is influenced by perceived usefulness and perceived ease of use; in the TPB, behavioral attitudes, subjective norms, and perceived behavioral control are three core concepts that work together to influence behavioral intentions and, ultimately, the behavior itself. In addition, the study points out that risk perception can have a positive effect on attitude toward use and intention to use; safety climate can have a positive effect on risk perception, attitude toward use and perceived usefulness; and self-efficacy has a positive effect on perceived ease of use. This constitutes the structural model of the 19 pathways shown in Figure 3. Theoretical framework of the extended TAM and TPB for VRTS usage.

## 3 Materials and methods

### 3.1 Study area and data collection

**Figure 4:**
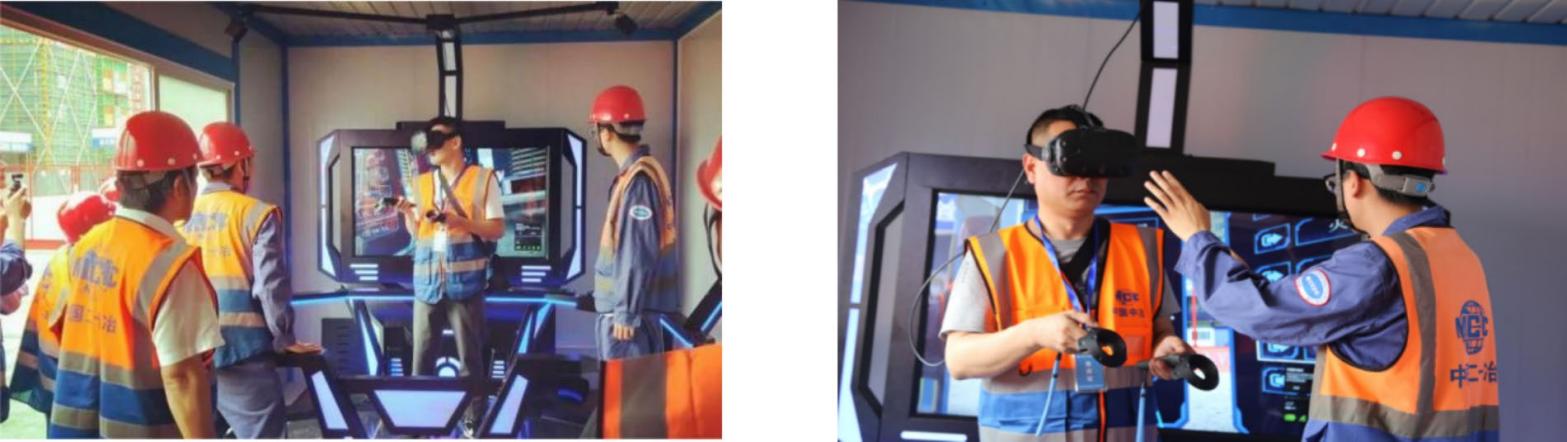
Field use of VRTS training by CWs.

VRTS has been widely applied in various pilot studies and applications in Shenzhen, China. In the process of selecting the sample, we employed stratified random sampling to ensure the diversity and representativeness of the sample. We stratified the construction workers based on the different construction projects they were involved in. Subsequently, within each stratum, we randomly selected a certain number of workers as the sample. This sampling strategy helps to capture the acceptance of VRTS among workers from different backgrounds and working environments, thereby making the research results more generalizable and applicable. In this survey, we randomly selected 528 CWs equipped with VRTS from different large-scale construction projects in Shenzhen to participate in this study (In the study, we displayed a photo depicting CWs using VRTS in the Dameisha-Xiaomeisha Branch Comprehensive Pipeline Corridor project, constructed by the Shenzhen branch of China Metallurgical Group Corporation, as shown in Figure 4).

In order to ensure the fairness and consistency of the experiments, in most cases, all subjects were required to use the same VRTS. Since VRTS is usually highly customizable, the setting of the training content can depend on the specific objectives of the training, which will minimize potential interference due to system differences. Prior to the commencement of the survey, workers were informed of their right to withdraw from the study, and it was assured that the collected data would be treated with anonymity and confidentiality. This consent process was approved as part of our study protocol by the Institutional Review Board. Before commencing the survey, we ensured that all methods were carried out in strict accordance with the guidelines and regulations set forth by the ethical standards of Jilin Jianzhu University. The experimental protocols, including the survey method and data collection process, received prior approval from Jilin Jianzhu University’s Institutional Review Board, ensuring adherence to ethical research practices.

**Figure 5:**
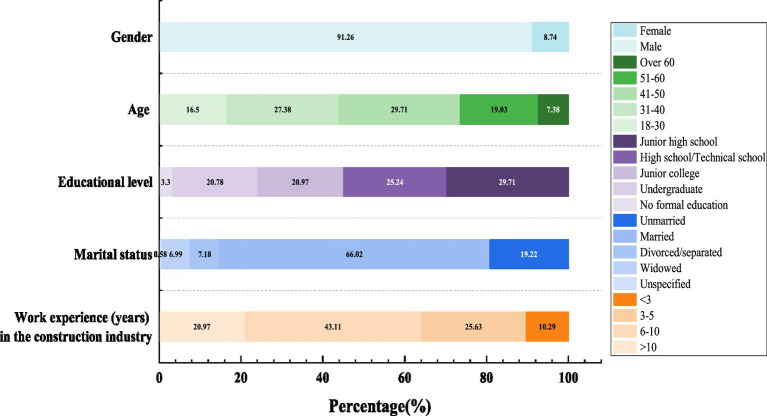
Participants’ demographic information (*n* = 515).

This paper conducts descriptive statistical analysis on the 515 collected survey questionnaires. Detailed results of descriptive statistical analysis are presented in Figure 5. The data findings reveal the following: (1) Gender ratio: 91.26% of respondents were male. (2) The age distribution survey revealed that the majority of respondents were middle-aged, between 31 and 60 years old. (3) Regarding educational level, most respondents had completed education up to the college level or below, with a relatively small percentage holding a bachelor’s degree or higher. (4) The research on marital status showed that the majority of respondents were married, accounting for 66.02% of the total. (5) The study on respondents’ working experience in the construction industry revealed that 43.11% had 6–10 years of experience, or more than 10 years of experience was relatively low. Having relevant working experience in the construction industry positively influenced the respondents’ cognitive acceptance of VRTs. These findings align with the current situation of construction workers in Shenzhen, China, which adds to the validity and reliability of the questionnaire data to a certain extent.

### 3.2 Questionnaire design

The questionnaire comprised two parts. The first part aims to gather background information about CWs, including their training-related background such as gender and education level. The second part involves a structured questionnaire that combines TAM and TPB to assess CWs’ acceptance of the VRTS. The details of all the questionnaire items are presented in Table 1. A five-point Likert scale ranging from 1 = ‘completely disagree’ to 5 = ‘completely agree’ is employed in this study to measure the intention of the scale variables.

**Table 1:** Summary of construction item statistics.

Construct	Item	Item description	Source
Safety climate (SC)	SC1	Construction enterprise will provide all the necessary equipment related to safety training regardless of cost.	(53, 79)
	SC2	When construction workers appear unsafe behavior (operation without safety training), site safety personnel will immediately intervene and order them to stop operation.	
	SC3	The safety staff of the site management promptly addresses and resolves any safety issues that arise, ensuring their long-term resolution.	
	SC4	The site management thinks that safety is more important than productivity.	
Self-efficacy (SE)	SE1	I believe I can handle virtual reality hardware very well.	(58, 80)
	SE2	I believe my performance in virtual reality to match that in real-life scenarios.	
	SE3	I believe VRTS can improve my operation level.	
Perceived usefulness (PU)	PU1	I believe that VRTS are more efficient than traditional trainings.	(29, 81)
	PU2	I believe using VR hardware would help improve my training experience.	
	PU3	I believe that VRTS could help me adapt workplace more quickly.	
	PU4	I believe that VRTS could help me reduce my unsafe behavior.	
Perceived ease of use (PEU)	PEU1	I believe the VR hardware is easy to use.	(29, 81)
	PEU2	I believe the content of VRTS is easy to understand.	
	PEU3	I believe that virtual reality training systems are flexible to interact with.	
Attitude toward learning VR content (ATT1)	ATT11	I am satisfied with the content of virtual reality training systems.	(29, 78, 82)
	ATT12	I prefer the content presented in a VR technology environment compared to the traditional way of presenting training content.	
	ATT13	The way the content is presented in the VR technology environment will make me more aware of the content.	
Attitude toward using VR-hardware (ATT2)	ATT21	I prefer to use VR hardware for interaction when conducting VR technology training.	
	ATT22	I am satisfied with the effectiveness of training using virtual reality hardware when conducting virtual reality technology training.	
	ATT23	I think using VR hardware is a wise choice when training in VR technology.	
Intention to use VR (IUV)	IUV1	I will accept VRTS within the foreseeable future.	(29, 78, 82)
	IUV2	I will recommend CWs to use virtual reality training systems.	
	IUV3	Using virtual reality training systems in the foreseeable future is important to me.	
Subjective norm (SN)	SN1	Your close family members, including parents, children, and spouse, would encourage your use of virtual reality training systems.	(16, 83, 84)
	SN2	Your coworkers or supervisor, who have an influence on you, would support the idea of using virtual reality training systems.	
	SN3	Your parents, children, and spouse, who hold significance in your life, would prefer that you should use VRTS.	
Perceived behavioral control (PBC)	PBC1	I can skillfully use virtual reality hardware.	(16, 20)
	PBC2	I have the resources, knowledge, and ability to use virtual reality.	
	PBC3	Using virtual reality hardware was entirely within my control.	
Risk-perception (RP)	How concerned or unsafe would you feel about potential negative consequences if you encounter the following work events or situations?		(43, 48)
	RP1	Get directly involved in the field without accept virtual reality training systems.	
	RP2	Without understanding the overall construction environment, directly participate in the site work.	
	RP3	Without using more experiential VR hardware when received VRTS.	
Actual VR usage (AVU)	AVU1	How many times will you use virtual reality training systems during a week?	
		1. Not at all.	
		2. About once a week.	
		3. 2–3 times a week.	
		4. 4–5 times a week.	
		5. About once a day.	
	AVU2	How many hours will you use virtual reality training systems every week?	
		1. <1 h	
		2. 1–5 h	
		3. 5–10 h	
		4. 10–15 h	
		5. >15 h	
	AVU3	How much time will you use before entering the site?	
		1. <10 min	
		2. 10–20 min	
		3. 20–30 min	
		4. 30–40 min	
		5. >40 min	

### 3.3 SEM and ANN

Structural equation modeling (SEM) is employed to evaluate this study, which involves a two-step process, measurement model evaluation and structural model evaluation (59–61). The first step involves the use of confirmatory factor analysis to assess the measurement model, focusing on assessing reliability and construct validity (encompassing discriminant and convergent validity). Specifically, convergent validity is assessed by calculating composite reliability (CR) and factor loading values, and discriminant validity is assessed by comparing the absolute value of the square root of the Average Variance Extracted (AVE) for the latent variable to the correlation coefficients between the other latent variables to ensure that the questionnaire can effectively reflect the questions to be researched. Another step involves performing structural model evaluation and estimating the structural relationships between the structures to test the hypotheses, using common fit metrics, e.g., X^2^/df, RMSEA, GFI, TLI, CFI, etc. to test the fit between the hypothesized theoretical model and the actual data to increase the persuasiveness of the model. Data analysis is carried out via SPSS 26.0 and AMOS 24.0 software.

The SEM approach has demonstrated substantial explanatory power in elucidating the linear relationships among factors in the acceptance model of VRTS for CWs. However, Jia Sim et al. (62) argue that such conventional statistical methods oversimplify the intricate decision-making processes of humans. The emergence of ANNs is perceived as capable of more accurately representing the potential nonlinear relationships among these variables and predicting the contributions of individual factors. Consequently, this study adopts a hybrid analysis method combining SEM and ANN in two stages, aiming to identify both linear and nonlinear relationships between structures, facilitating a more precise modeling and understanding of complex data relationships.

## 4 Results

### 4.1 Analysis of factors influencing CWs’ acceptance based on SEM

Firstly, a reliability test is conducted using Cronbach’s alpha to assess the internal consistency reliability of the scale (63), with a predefined threshold of 0.7. As shown in Table 2, the Cronbach’s alpha values of all constructs are between 0.753 and 0.880, and the reliability of each dimension of the variables reaches above 0.7, demonstrating the high reliability of the questionnaire and its design.

**Table 2:** Correlations among constructs.

	RP	SC	SE	PU	PEU	ATT1	ATT2	IUV	AVU	SN	PBC
RP	0.769										
SC	0.438	0.81									
SE	0.393	0.294	0.797								
PU	0.429	0.294	0.374	0.776							
PEU	0.334	0.275	0.336	0.329	0.789						
ATT1	0.433	0.363	0.448	0.518	0.286	0.765					
ATT2	0.319	0.254	0.42	0.377	0.261	0.385	0.754				
IUV	0.499	0.486	0.523	0.48	0.359	0.543	0.437	0.714			
AVU	0.392	0.426	0.432	0.459	0.312	0.408	0.252	0.575	0.799		
SN	0.413	0.381	0.395	0.358	0.287	0.444	0.232	0.56	0.477	0.826	
PBC	0.289	0.242	0.276	0.218	0.166	0.267	0.205	0.674	0.32	0.332	0.826

Next, the paper conducts structural validity tests. Factor analysis is a commonly used method to assess structural validity, which is further confirmed through convergent validity and discriminant validity. Convergent validity assesses the consistency of different measures when evaluating the same constructs. To verify, it is recommended that each construct has a composite reliability (CR) value and factor loading value exceeding 0.7, and an average variance extracted (AVE) value greater than 0.5 (59). The results of the convergent validity test are shown in Table 3. All principles are satisfied, indicating good convergent validity of the questionnaire data. Discriminant validity refers to the extent of a test to differentiate between distinct factor traits (64). The technique is utilized to establish the discriminant validity of the measure by comparing the square root of the AVE of the latent variable with the absolute value of the correlation coefficient between the other latent variables (59), and the results of the test for discriminant validity are shown in Table 2. The square root of the AVE for all latent variables exceeds the absolute value of the correlation coefficients between each latent variable, indicating sound discriminant validity among the constructs. Hence, the structure of the model in this study is established with scientific rigor and logical coherence.

**Table 3:** Reliability and convergent validity assessment results.

Construct	Item	Mean	Standard deviation	Factor loading	AVE	CR	Cronbach’ s alpha
Risk perception (RP)	RP1	3.54	1.339	0.805	0.592	0.812	0.810
	RP2	3.58	1.307	0.685			
	RP3	3.28	1.236	0.811			
Safety climate (SC)	SC1	3.17	1.452	0.630	0.656	0.882	0.880
	SC2	3.45	1.359	0.902			
	SC3	3.52	1.374	0.765			
	SC4	3.55	1.366	0.910			
Self-efficacy (SE)	SE1	3.49	1.390	0.703	0.635	0.838	0.835
	SE2	3.83	1.373	0.894			
	SE3	3.64	1.341	0.782			
Perceived usefulness (PU)	PU1	3.86	1.237	0.508	0.602	0.850	0.840
	PU2	3.79	1.147	0.892			
	PU3	3.77	1.156	0.632			
	PU4	3.30	1.377	0.977			
Perceived ease of use (PEU)	PEU1	3.70	1.336	0.863	0.622	0.830	0.824
	PEU2	3.59	1.301	0.649			
	PEU3	3.72	1.369	0.837			
Attitude toward VR content (ATT1)	ATT11	3.59	1.322	0.706	0.585	0.807	0.798
	ATT12	3.83	1.356	0.708			
	ATT13	3.58	1.597	0.869			
Attitude toward VR hardware (ATT2)	ATT21	3.67	1.331	0.837	0.569	0.796	0.786
	ATT22	3.52	1.523	0.788			
	ATT23	3.55	1.358	0.620			
Intention to use VR (IUV)	IU1	3.36	1.503	0.761	0.510	0.757	0.753
	IU2	3.60	1.508	0.738			
	IU3	3.32	1.500	0.638			
Actual VR-usage (AVU)	AVU1	3.44	1.398	0.766	0.638	0.840	0.835
	AVU2	3.65	1.437	0.863			
	AVU3	3.58	1.617	0.763			
Subjective norm (SN)	SN1	3.54	1.552	0.934	0.683	0.865	0.856
	SN2	3.22	1.616	0.798			
	SN3	3.56	1.528	0.734			
Perceived behavioral control (PBC)	PBC1	2.98	1.371	0.873	0.682	0.865	0.863
	PBC2	3.06	1.420	0.775			
	PBC3	3.08	1.379	0.827			

And the next step is to conduct fitness tests on the evaluation model; x^2^/df, RMSEA, GFI, TLI, CFI, and IFI.

Absolute fitting index: This type of indicator directly measures the difference between the model and actual data. The smaller the value, the better the fit of the model.

Chi square/degree of freedom ratio (X^2^/df): This ratio is used to evaluate the fit of the model. Generally speaking, a ratio of less than 3 is considered acceptable, while a ratio of less than 5 indicates a good fit of the model. RMSEA: The sum of squared residuals divided by the square root of the model’s degrees of freedom. If the RMSEA value is less than or equal to 0.05, it is considered that the model has a good fit; If it is less than or equal to 0.08, it is considered that the fit of the model is still acceptable.

Incremental fitting index: This type of indicator compares the fitting degree of the benchmark model and the target model. The larger the value, the better the fitting degree of the model. CFI (Comparative Fit Index): The closer the value is to 1, the better the fitting effect of the model. If the CFI value is greater than or equal to 0.9, it is considered that the model has a good fit.

Simplicity fitting index: This type of indicator evaluates the degree of simplicity of the model, that is, the simplicity of the model parameters while maintaining the model fitting degree. GFI (Goodness of Fit Index): a correction to the chi square value, which reflects the difference between observed data and theoretical models. The closer the value is to 1, the better the model fitting effect. TLI (Tucker Lewis Index): The closer the value of TLI is to 1, the better the model fitting effect. When TLI ≥ 0.90, the model fitting effect is usually good. A value greater than 0.80 is acceptable. The model fit index values and the requirements to meet the recommendations are shown in Table 4, indicating a satisfactory fit index of the model.

**Table 4:** Structural model assessment results.

Model fit index	Value	Recommended value	Result
X^2^/df	1.589	<3	Acceptable
RMSEA	0.034	<0.08	Acceptable
GFI	0.918	>0.80	Acceptable
TLI	0.962	>0.90	Acceptable
CFI	0.968	>0.90	Acceptable
IFI	0.968	>0.90	Acceptable

**Figure 6:**
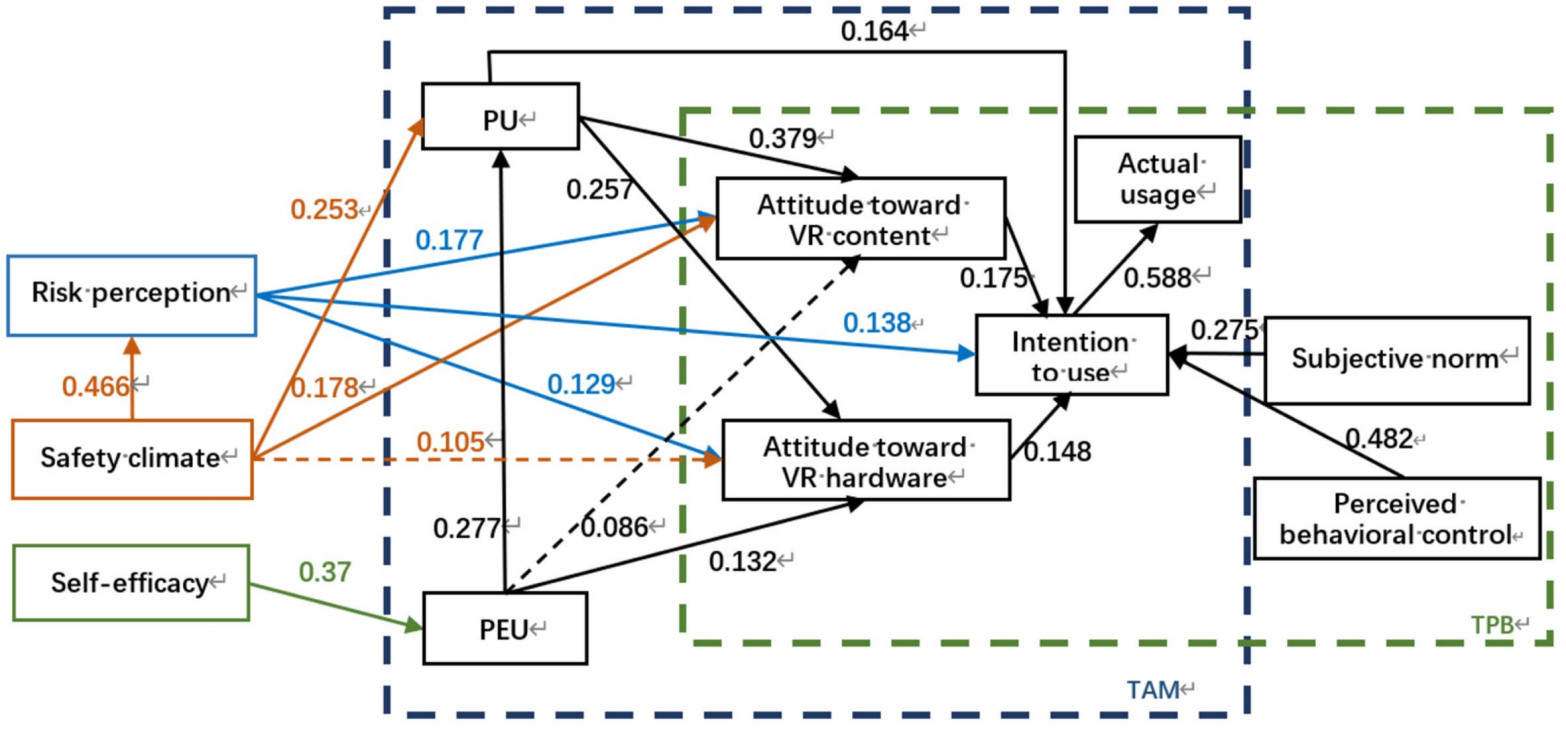
Results of structural model evaluation where the median value is the standardized path coefficient (dashed line indicates no significance, solid line indicates significance, and mathematical relationships such as). PU = 0.277 × PEU.

Based on the above results, the relationship between each variable by the path coefficient and its significance is tested. The results of model validation are obtained as shown in Table 5 below, and the findings indicates that 15 hypotheses are statistically significant except for hypotheses H5 (Perceived ease of use has a positive impact on the attitude toward learning VR content) and H17 (Safety climate has a positive effect on attitude of using VR hardware). PEU positively influenced the attitudes of PU and CWs toward using VR hardware. Following the obtained results, the significance of the relationship between each variable using path coefficients is assessed. The ATT, SN, and PBC of CWs positively influence their willingness to use the VRTS. The willingness of CWs to use the VRTS has a positive impact on their actual usage. RP has a positive influence on both CWs’ attitudes toward the VRTS and their willingness to use them. SC has a significant positive effect on PU, CWS’ attitudes toward VR content and RP. These results are shown in Figure 6.

**Table 5:** Hypotheses testing results.

Hypothesis	Standardized path coefficient	*p*-value	Result
H1. Perceived ease of use has a positive effect on perceived usefulness.	0.277	<0.001	Supported
H2. Perceived usefulness has a positive effect on attitude of VR content.	0.379	<0.001	Supported
H3. Perceived usefulness has a positive effect on attitude of using VR hardware.	0.257	<0.001	Supported
H4. Perceived usefulness has a positive effect on the intention to use.	0.164	<0.001	Supported
H5. Perceived ease of use has a positive effect on attitude of VR content.	0.086	0.074	Not supported
H6. Perceived ease of use has a positive effect on attitude of using VR hardware.	0.132	0.013	Supported
H7. Attitude of VR content has a positive effect on the intention to use.	0.175	<0.001	Supported
H8. Attitude of using VR hardware has a positive effect on the intention to use.	0.148	0.001	Supported
H9. Subjective norm has a positive effect on intention to use.	0.275	<0.001	Supported
H10. Perceived behavioral control has a positive effect on intention to use.	0.482	<0.001	Supported
H11. Intention to use has a positive effect on actual usage.	0.588	<0.001	Supported
H12. Risk perception has a positive effect on attitude of using VR content.	0.177	0.001	Supported
H13. Risk perception has a positive effect on attitude of using VR hardware.	0.129	0.029	Supported
H14. Risk perception has a positive effect on the intention to use.	0.138	0.002	Supported
H15. Safety climate has a positive effect on perceived usefulness.	0.253	<0.001	Supported
H16. Safety climate has a positive effect on attitude of using VR content.	0.178	<0.001	Supported
H17. Safety climate has a positive effect on attitude of using VR hardware.	0.105	0.072	Not supported
H18. Safety climate has a positive effect on risk perception.	0.466	<0.001	Supported
H19. Self-efficacy has a positive effect on perceived ease of use.	0.371	<0.001	Supported

### 4.2 Importance analysis of factors influencing CWs’ acceptance based on ANN

**Figure 7:**
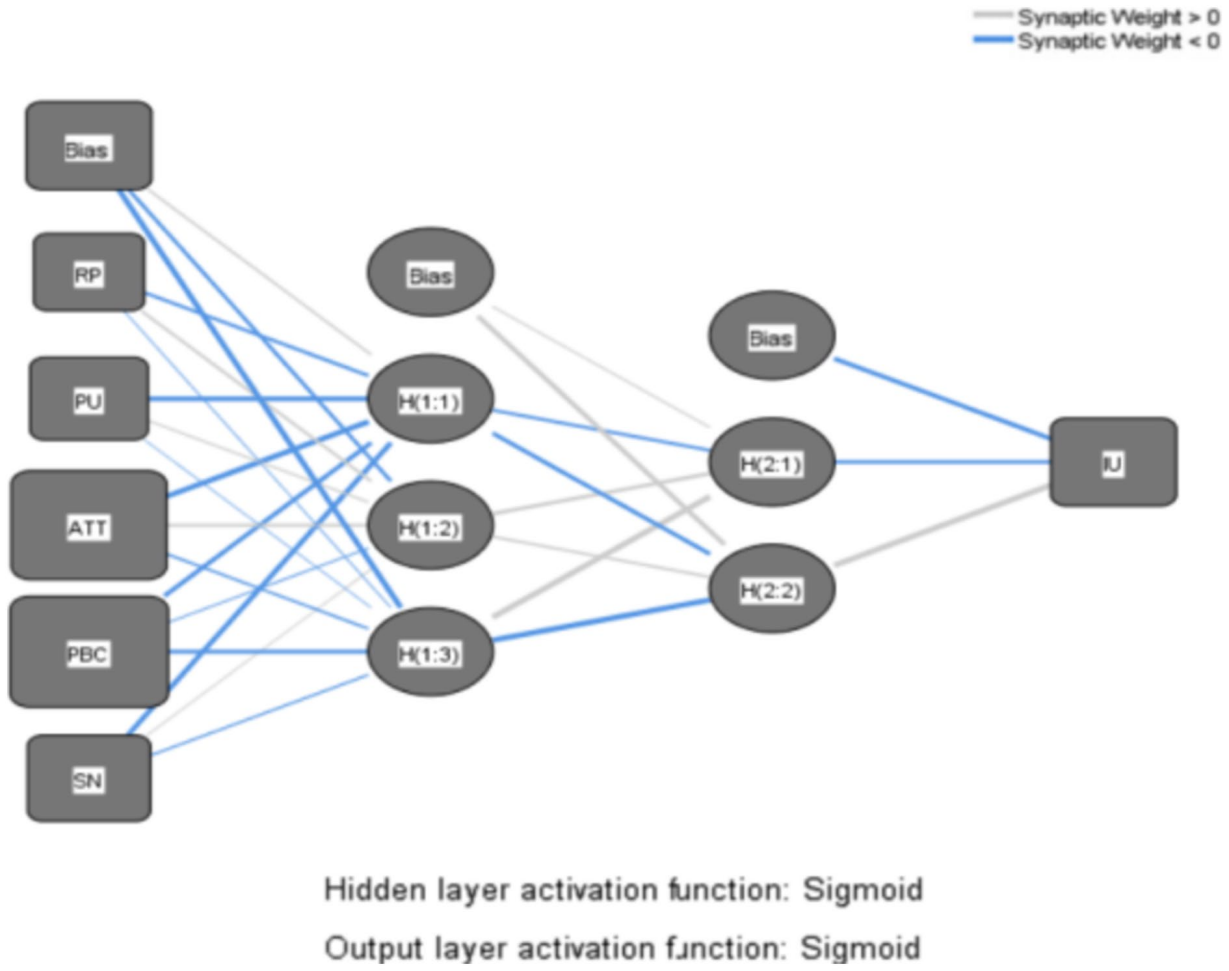
ANN Model A.

**Figure 8:**
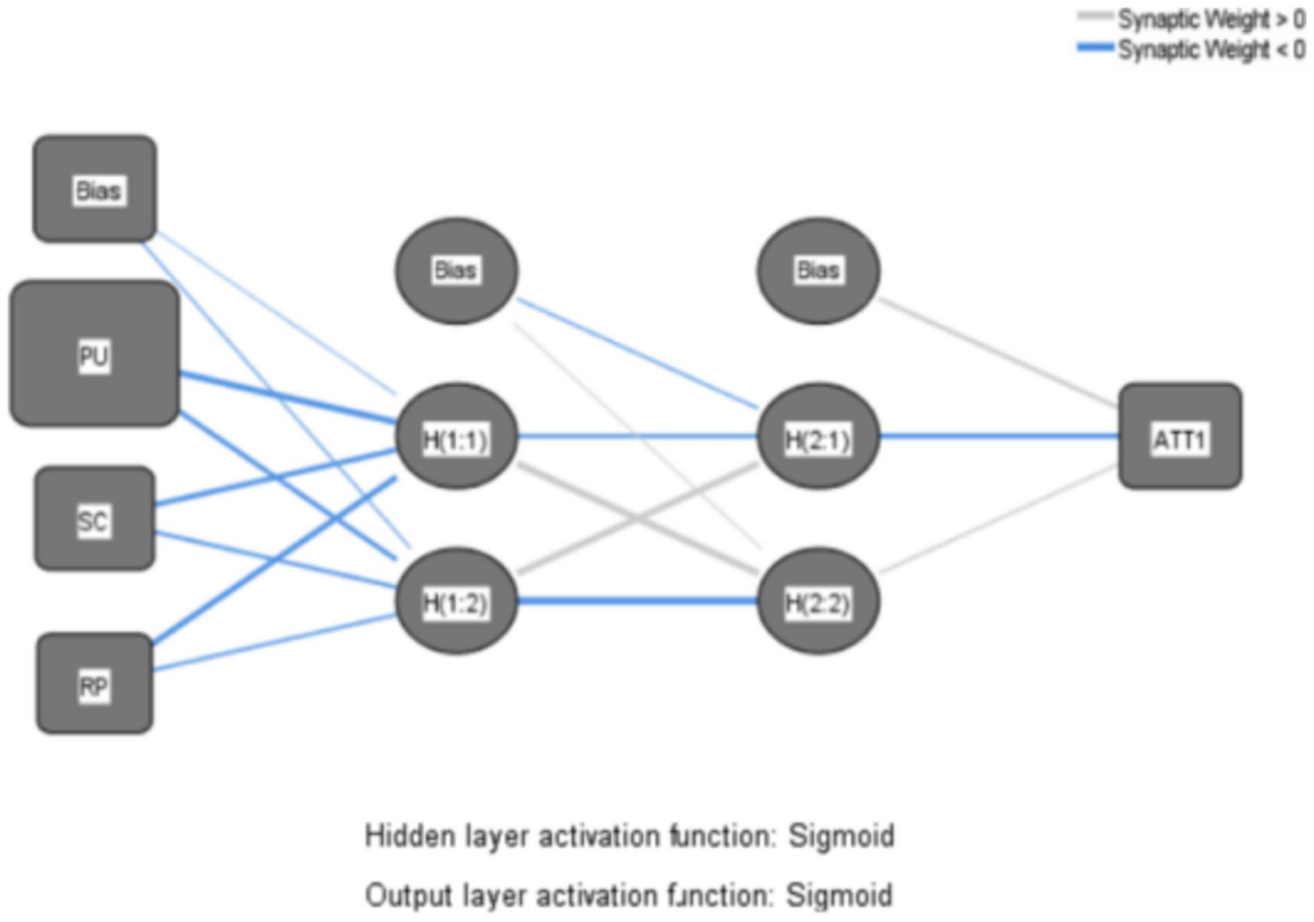
ANN Model B.

**Figure 9:**
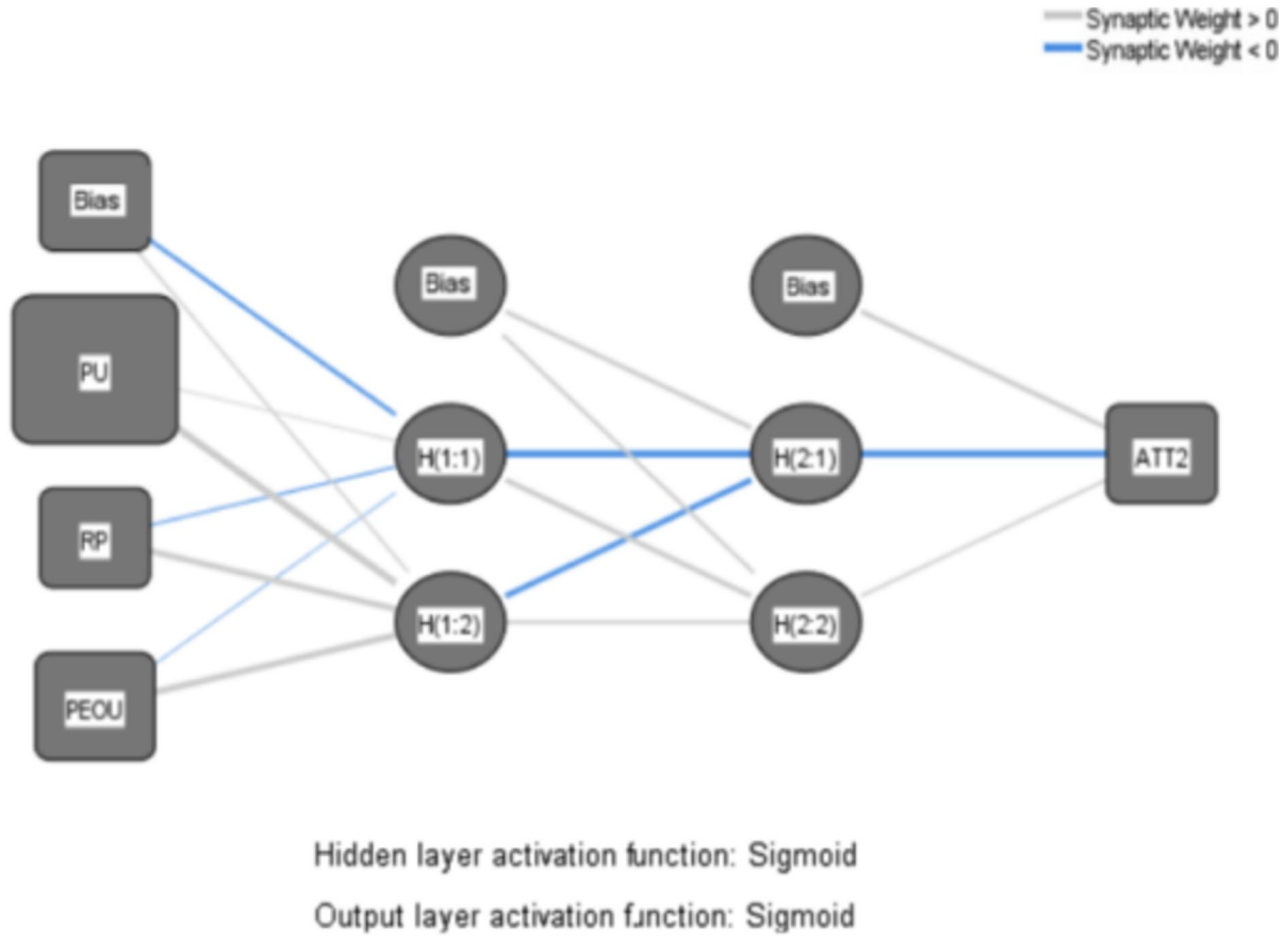
ANN Model C.

The primary steps of the research are outlined as follows: First, based on the path analysis results derived from SEM, three distinct ANN models are constructed, illustrated in Figures 7–9, to depict the intricate nonlinear relationships between construction workers’ attitudes and willingness to use VRTS and various variables, thereby delving further into the underlying mechanisms of their acceptance of the system. Next, the root mean square errors (RMSE) of the 10 networks are computed to evaluate the predictive accuracy of the generated ANN models. Lastly, sensitivity analyses are conducted on the ANN models to ascertain the importance of each input variable to the output variable, thereby elucidating the factors crucial to the acceptance of the VRTS among CWs.

Firstly, models A, B, and C are subjected to scrutiny under an Artificial Neural Network (ANN) Multilayer Perceptron framework. To enable the ANN model to exhibit nonlinearity, the Sigmoid function is employed as the activation function in both the hidden and output layers. Furthermore, to enhance the accuracy of the nonlinear model (65). And a two-layer deep ANN is adopted in the hidden layer in order to mitigate the issue of overfitting, a 10-fold cross-validation method is employed using a deep ANN Multilayer Perceptron algorithm (66). This involves utilizing 90% of the data for the training phase, while reserving the remaining 10% for testing. The maximum number of iterations is set at 5,000, with a learning rate of 0.05.

Subsequently, the Root Mean Square Error (RMSE) is calculated for 10 network sets each comprising training and testing datasets, to assess the predictive accuracy of the ANN models. A lower RMSE value, closer to zero, signifies a better fit of the model and higher accuracy. The RMSE results for each of the three models across their respective 10 neural networks are presented in Table 6.

**Table 6:** RMSE values of the ANN Model A-C.

Network	Model A		Model B		Model C	
	RMSE (training)	RMSE (testing)	RMSE (training)	RMSE (testing)	RMSE (training)	RMSE (testing)
1	0.412	0.361	0.389	0.376	0.327	0.339
2	0.385	0.354	0.358	0.368	0.321	0.385
3	0.353	0.342	0.335	0.347	0.347	0.339
4	0.406	0.363	0.374	0.367	0.308	0.372
5	0.378	0.364	0.372	0.356	0.272	0.366
6	0.383	0.388	0.366	0.393	0.343	0.314
7	0.405	0.340	0.351	0.404	0.326	0.353
8	0.369	0.363	0.388	0.386	0.281	0.336
9	0.376	0.372	0.423	0.355	0.287	0.329
10	0.364	0.356	0.397	0.368	0.314	0.369
Mean	0.383	0.360	0.375	0.372	0.313	0.350
SD	0.018	0.013	0.024	0.017	0.026	0.033

The reported test and training values, all ranging between 0.2 and 0.5, adhere to empirical rules suggesting that the models can predict data with considerable accuracy. Moreover, the deep Multilayer Perceptron of the ANN achieved an average accuracy of 87.34% during Model A’s training phase, which was closely matched by an 87.22% average accuracy in testing. For Model B, it yielded an average training accuracy of 79.04%, dropping slightly to 77.83% in testing. Similarly, Model C also demonstrated an average training accuracy of 79.04%, with a test accuracy of 77.83%. These outcomes substantiate the exceptional performance of the ANN as a supervised nonlinear classification algorithm via its Multilayer Perceptron in forecasting the utility of perceptions.

Lastly, to elucidate the disparities within the inherent structure and to gauge the relative significance of predictor variables, sensitivity analysis was conducted. This involved analyzing the normalized importance percentages of the explanatory variables to gauge each factor’s contribution. The subsequent results of the relative importance analysis for the input variables across the three neural network models are presented in Table 7. This analysis provides insights into how each variable influences the prediction outcomes, thereby enhancing our understanding of their predictive power within the context of the models.

**Table 7:** Sensitivity analysis for the ANN Model A–C.

	Influences	Mean importance	Normalized importance	Sequence
Model A	PBC	0.462	100.00%	1
	SN	0.204	44.20%	2
	ATT1	0.125	27.10%	3
	RP	0.086	18.60%	4
	PU	0.068	14.80%	5
	ATT2	0.055	11.80%	6
Model B	PU	0.536	100.00%	1
	SC	0.251	39.90%	2
	RP	0.214	46.50%	3
Model C	PU	0.478	100.00%	1
	PEU	0.242	50.60%	2
	RP	0.280	58.70%	3

## 5 Discussion and policy implications

This paper explores the acceptance of VRTS among CWs by combining the extended TAM and TPB with RP, SC, and SE. Data from 528 CWs were collected in Shenzhen to test and empirically validate. The results show that CWs have moderately high attitudes and IU to VRTS. While previous studies explored the advantages and effectiveness of VRTS in construction fields (6, 67, 68), which had been proved as the limited research focusing on the actual willingness and acceptance of CWs in order to advance the application of virtual reality training and explore the associated behavior-driven mechanisms. Conducting this research in Shenzhen (a highly developed city situated in the southern coastal region of China, where the implementation and popularity of VRTS are relatively high) will bridge the existing research gap.

### 5.1 CWs’ acceptability and determinants

In this paper, TAM and TPB are combined for the first time and three external influences, RP, SC and SE, are included to construct a model of construction workers’ acceptance of VRTS, and has tests and empirical researches on the obtained 515 valid questionnaires.

This study shows that the acceptance of VRTS training by CWs shows a more positive trend. First of all, this paper observes higher mean scores in the questionnaire results, e.g.: Mean AVU = 3.56, Mean ATT1 = 3.66, Mean IU = 3.43, Mean ATT2 = 3.58, and they are in the medium-high range, which is in line with the results of the previous study consistent with previous findings that VR-based training is more popular (12). These findings indicate a notably positive tendency among Chinese construction workers toward the adoption of VRTS, concurrently reflecting a level of acknowledgment by the workers, as the primary beneficiaries, of the system’s advantages and effectiveness within the construction sector. This recognition underscores the significant potential for VR technology-based safety training systems to be implemented and popularized within China’s construction industry, thereby enhancing safety training practices and contributing to a safer working environment. The discovery holds substantial implications for promoting the application of advanced technologies in addressing occupational safety challenges, fostering a culture of safety awareness, and ultimately improving safety standards across the construction landscape in China.

In the part of TAM, we find that, firstly, PU has a significant positive effect on ATT and IU, which plays a key role in determining whether CWs use VRTS or not and contradicts the findings of Ming et al. Their hypothesis that PU has a positive effect on IU was rejected, and who argued that CWs would not use VR training just to improve the effectiveness of the training (12). The reason for this difference may be that the CWs who participated in this study used VR training with the addition of VR hardware, which increased PU. Thus, this paper argues that the higher the PU, the more likely it is that CWs will recognize the potential benefits of VRTS and be willing to try it out to be more productive. Next, the hypothesis that PEU has a positive impact on ATT1 is not valid, which is inconsistent with Davis’s hypothesis in TAM, and this result suggests that even though the VRTS about the content presentation is convenient, CWs still face some difficulties in understanding the VR content, which is due to the fact that at this stage, the cultural level of the CWs in China is still limited, and they have been in a long period of time in the traditional way of training with the help of trainers’ explanations. This conclusion is similar to the research content of C. Shi et al. VRTS will be introduced into the Chinese construction field as a new product and is in the growth stage of the product lifecycle, and the vast majority of CWs are still in the wait-and-see stage of its use (69), and its development is bound to be a long term process, and it is not possible to completely replace the traditional training methods in a single step. However, this is contrary to the belief of Estefany that VR training could replace traditional training, which also shows that there is a great gap in the development of VRTS in different countries (70). At this stage, we can integrate VRTS with traditional training and gradually lead them to shift to the training model of VRTS. The aim is to help CWs better adapt to and accept the VRTS training method, so as to promote the steady growth and development of VRTS in the Chinese market, and then bring innovative changes and progress to the training industry. Except for the hypothesis of PU on ATT1 mentioned above, the rest of the results are consistent with Davis’ hypothesis in TAM that PEU positively affects PU, and IUs using VRTS are positively affected by ATT, and ATT1 is more significant in positively affecting IUs than ATT2, which is a very important finding. It suggests that CWs perceive the acquisition of knowledge during the training process as a better way to enhance individuals’ operation on construction sites to reduce the occurrence of their own unsafe behaviors than the immersion brought by hardware devices. This finding also reminds VRTS designers that they cannot focus solely on the performance and technology of the hardware and neglect the importance of the content.

For the TPB component, intention to use VRTS is positively influenced by ATT, SN, and PBC, a result consistent with the TPB presented by Ajzen. Among these three drivers, PBC has the most significant positive effect on IU (16). Similar findings are obtained in the acceptance study on Siu Shing Man et al. PBC has the greatest impact on the acceptance of construction workers’ PPE (19). This suggests that when CWs perceive that their current level of operational competence and knowledge will enable them to achieve the desired results when training with VRTS, their IU will subsequently increase, which in turn will drive them to participate in the training more actively and conscientiously in order to achieve the best results. In contrast, the positive effect of SN on IU is slightly less significant, but it is worth noting that this result suggests that more and more companies and organizations are beginning to recognize the potential of VRTS technology for training, and that they are encouraging CWs to learn and master this technology.

The model of CWs’ acceptance of VRTS training constructed in this paper considers three external influences in addition to the combination of TAM and TPB described above. Firstly, RP positively affects both ATT and IU, whereas the dimension of risk perception has not been considered in previous studies on VR training acceptance (18, 19), which shows that when construction workers perceive that they have not been trained in VRTS and the risk of entering the site, they become more cautious and vigilant. This enhanced perception will motivate them to be more active in finding and using tools or techniques that can reduce risk, i.e., increase their attitude and willingness to use VRTS. Secondly, the dimension of SC is considered in the model of construction workers’ acceptance of VRTS training, and the positive effect of SC on PU is significant, consistent with the findings of Xiaowen Hu et al. They report that organizational safety climate positively influences perceived usefulness of the risk-awareness procedure among miners (71). In addition, the positive effect of SC on RP is similarly significant, which is consistent with Bhavana et al.’s finding that safety climate can increase risk perception among US construction workers (53). In previous studies, safety climate tends to have a positive effect on attitudes (20, 72). In contrast, the positive effect of SC on attitudes is only partially validated in this study, with a positive effect holding for ATT1, but no significant positive effect for ATT2. After in-depth analysis, this paper finds that the safety climate of construction enterprises (Mean SC = 3.42) is at a medium-high level, which to a certain extent reflects the in-depth understanding and practice of safety training in construction enterprises. However, at present, China’s CWs have not yet fully realized the transformation from the traditional training mode to the VRTS training mode, and they still believe that the learning of theoretical knowledge is more important, and that the acquisition of knowledge is an effective way to improve their safety perception. While VRTS-based safety trainings not only focus on the transfer of theoretical knowledge, but also strengthen the training of practical operation. We predict that the SC’s assumptions about ATT2 will be gradually verified over time. Currently CWs are more influenced by PUs and PEUs for ATT2, and if the hardware itself suffers from insufficient performance, complexity of operation, or poor comfort, CWs may have reservations about the use of VR hardware, even in an environment with very high SC. Finally, external incentives on the ease of use of VRTS have rarely been investigated, this study introduces SE as an external incentive, and we find that SE has a significant positive effect on PEU. And the SE of CWs (Mean SE = 3.65) is above average, which indicates that they have strong control over the VRTS, and this higher self-confidence not only promotes their acceptance of the VRTS, but also motivates them to proactively explore new features and application scenarios of the VRTS.

### 5.2 Implications

Firstly, this study broadens the field of VR training researches. Current research on VR technology training mainly focuses on the validation of effectiveness, while the acceptance of CWs has rarely been explored. This paper aims to more accurately capture the acceptance of CWs to VRTS by establishing a theoretical framework explaining the acceptance of VRTS, adopting structural equation modeling to analyze the linear relationship between the factors in the theoretical model, and using attitudes and behavioral intentions. The results of the questionnaire completed by the CWs show a high level of acceptance of the VRTS, indicating that CWs believes that the immersive experience provided by the VRTS not only allows them to conduct hands-on training in a safe environment, but also helps to improve their safety awareness and skill levels. This finding lays a certain foundation for promoting VRTS within China.

Secondly, the conclusions drawn from this study will provide strong policy guidelines and recommendations to support the promotion of the widespread use of VRTS in the construction industry. (1) On the enterprise side: the hypothesis of H5 does not reflect the fact that CWs in China have been in traditional training for a long time. And they believe that a better way to acquire knowledge is the explanation of trainers, which requires construction enterprises to make a good transition from traditional VRTS, not only to equip professional safety trainers to explain the content shown in VRTS, but also to evaluate and summarize the CWs’ performances in this virtual environment and give timely feedback. SC is an external influence that positively affects PUs, RPs, and ATTs1, so to promote the sustainable development of VRTS, enterprises must be committed to improving the level of SC. In other words, enterprises must take the enhancement of safety culture as one of their core tasks, and improve the safety awareness of CWs through continuous optimization of safety management measures (regular safety inspections, creation of a safer working environment, etc.) and enhancement of the publicity based on VRTS safety training to promote the enhancement of utilization rates. In addition, operators need extra time to familiarize themselves with and master the operation and application of VRTS. Enterprises should design training programs to minimize disruption to operators’ daily work. A phased training approach can be adopted to allow employees to learn and adapt to the new system gradually without affecting the production schedule. (2) On the part of VRTS designers: SE has a positive impact on PEU. And PEU has a positive impact on ATT2. Increased SE promotes successful technology acceptance (73), and studies have shown that pleasure has a positive effect on SE (74, 75). Therefore, designers may consider gamifying the scenarios of accident scene simulations in VRTS to increase CWs’ sense of enjoyment and willingness to use virtual reality technology (76–78). Meanwhile, presenting the same VR content on different devices may produce different training effects. Therefore, when designers enhance and improve VR content, they should also consider the design elements of VR hardware, and strive for a seamless combination of content and hardware to achieve an immersive experience.

(3) Government level: At the governmental level: VRTS, as a new training model, often involves multiple factors such as equipment procurement, talent introduction, and equipment maintenance when implemented in construction enterprises, leading to a significant increase in operational costs. As a result, in many cities, the adoption of VRTS by some small construction enterprises is still limited due to economic cost considerations to promote the popularization of this technology, local governments should provide active financial subsidies and organize visits for construction companies to those that have successfully implemented integrated VRTS. Such initiatives can help enterprises recognize the returns on long-term investments, such as enhanced safety and reduced accident costs. Additionally, the government should offer cost-sharing or leasing options for small and medium-sized enterprises to alleviate their financial pressure. Furthermore, some enterprises may lack the infrastructure and technical knowledge to support the implementation of VRTS. To address this challenge, the government can conduct technology assessments, review existing VRTS systems to assist construction enterprises in identifying the need for upgrades to their systems, and provide necessary technical training or support services to help them overcome technological barriers.

Finally, the study also has a certain social impact. The construction industry is one of the important pillars of the national economy, which is important for promoting economic development and improving people’s livelihood. Improving the use of CWs on VRTS increases the level of safety awareness and skills, which not only reduces the occurrence of safety accidents, but also improves the quality and efficiency of construction projects and promotes the sustainable development of the construction industry. Therefore, the research in this paper not only has academic value, but also has far-reaching social significance.

## 6 Conclusion

The study, through a two-stage analysis employing SEM-ANN on an established theoretical model, provides in-depth insights into the current level of acceptance of VRTS among construction workers in China, along with pertinent underlying factors. It further outlines strategies and recommendations from technological, corporate, and governmental perspectives. The research yields the following conclusions and achievements.

Validation of Theoretical Models: Our findings endorse the applicability of both Technology Acceptance Model (TAM) and Theory of Planned Behavior (TPB) in examining the acceptance of VRTS by construction workers. A key revelation is that Perceived Ease of Use (PEU) significantly positively influences attitudes toward using VR hardware, suggesting a distinction between attitudes toward VR content and hardware is meaningful. This finding implies that, at present, VRTS should not fully replace conventional safety training in China but be integrated effectively to form a complementary and reinforcing training approach.

Importance of RP, SC, and SE: The research underscores the significance of Reward (RP), Social Influence (SC), and Self-Efficacy (SE) in shaping CWs’ attitudes and intentions toward using VRTS. RP positively impacts both usage attitude and intention, highlighting that awareness of risks associated with forgoing VR-based safety training enhances caution. Counter to expectations, high SC does not guarantee positive attitudes toward VR hardware when the hardware itself presents issues like poor performance or discomfort. Meanwhile, SE significantly enhances PEU, implying that fostering self-efficacy within VRTS encourages exploration and thereby increases perceived ease of use and overall acceptance.

ANN Model Performance and Insights: By segmenting SEM path analysis results into distinct ANN models, complex nonlinear relationships between variables were elucidated, with root mean square errors ranging from 0.2 to 0.5, evidencing a good fit. These models prove effective in predicting CWs’ attitudes and intentions. Sensitivity analysis identified Perceived Behavioral Control as a pivotal factor for usage intention and Perceived Usefulness as critical for usage attitude. Recognizing these, tailored policies and suggestions from design, corporate, and policy angles were formulated to facilitate the effective implementation of VRTS in the construction sector.

These findings contribute to the feasibility assessment of VRTS investment by construction firms, laying a theoretical groundwork for wider VRTS deployment. Ultimately, they aim to promote sustained VRTS usage among workers, thereby reducing unsafe behaviors and preventing potential accidents in the construction industry.

## 7 Limitations and future research

Despite the remarkable results of this study, there are limitations that need to be recognized. Firstly, the study focuses primarily on the external motivations of RP, SC and SE influencing CWs’ acceptance of VRTS and fails to adequately consider variables such as playfulness and emotional stimulation. Secondly, the respondents’ industry roles are limited to CWs, excluding other important roles and identities in the construction industry. This may affect the generalizability and applicability of the findings, as different roles and identities may have different perceptions and acceptance of VRTS. In addition, the single location of the questionnaire survey is limited to Shenzhen, China, and CWs from different cities or countries may have different perspectives on VRTS. Therefore, these findings may not be applicable to the construction industry in other regions or countries.

In future studies, it is recommended that the scope of the study be further expanded to cover more variables and influencing factors in order to gain a more comprehensive and in-depth understanding of the intrinsic and extrinsic motivations of CWs’ acceptance of VRTS. Meanwhile, in order to eliminate single-source bias, it is recommended that the sample source and coverage be expanded by inviting CWs from different cities and countries to participate in the survey, so as to obtain more diversified and comprehensive data. Consideration can also be given to expanding the scope of the study to other important players in the construction industry, such as architects, engineers, project managers, etc., in order to understand their perceptions and acceptance of VRTS. This will help promote the further development and application of VRTS technology and enhance the productivity and quality level of the construction industry.

## Data Availability

The raw data supporting the conclusions of this article will be made available by the authors without undue reservation.

## References

[1] Rokooei S Shojaei A Alvanchi A Azad R Didehvar N. Virtual reality application for construction safety training. Saf Sci. (2023) 157:105925. doi: 10.1016/j.ssci.2022.105925

[2] Sousa V Almeida NM Dias LA. Risk-based management of occupational safety and health in the construction industry – part 1: background knowledge. Saf Sci. (2014) 66:75–86. doi: 10.1016/j.ssci.2014.02.008

[3] Sawacha E Naoum S Fong D. Factors affecting safety performance on construction sites. Int J Proj Manag. (1999) 17:309–15. doi: 10.1016/S0263-7863(98)00042-8

[4] Demirkesen S Arditi D. Construction safety personnel's perceptions of safety training practices. Int J Proj Manag. (2015) 33:1160–9. doi: 10.1016/j.ijproman.2015.01.007

[5] Burke MJ Sarpy SA Smith-Crowe K Chan-Serafin S Salvador RO Islam G. Relative effectiveness of worker safety and health training methods. Am J Public Health. (2006) 96:315–24. doi: 10.2105/AJPH.2004.059840, PMID: 16380566 PMC1470479

[6] Sacks R Perlman A Barak R. Construction safety training using immersive virtual reality. Constr Manag Econ. (2013) 31:1005–17. doi: 10.1080/01446193.2013.828844

[7] He X Yuhua Z Qaidi S Isleem HF Zaid O Althoey F. Mine tailings-based geopolymers: a comprehensive review. Ceram Int. (2022) 48:24192–212. doi: 10.1016/j.ceramint.2022.05.345

[8] Akbaş A Marszałek W Kamieniarz A Polechoński J Słomka KJ Juras G. Application of virtual reality in competitive athletes - a review. J Hum Kinet. (2019) 69:5–16. doi: 10.2478/hukin-2019-0023, PMID: 31666884 PMC6815076

[9] Becerik-Gerber B Lucas G Aryal A Awada M Bergés M Billington S. The field of human building interaction for convergent research and innovation for intelligent built environments. Sci Rep. (2022) 12:22092. doi: 10.1038/s41598-022-25047-y, PMID: 36543830 PMC9769481

[10] Albert A Hallowell Matthew R Kleiner B Chen A Golparvar-Fard M. Enhancing construction hazard recognition with high-fidelity augmented virtuality. J Constr Eng Manag. (2014) 140:04014024. doi: 10.1061/(ASCE)CO.1943-7862.0000860

[11] Guo HL Li H Li V. VP-based safety management in large-scale construction projects: a conceptual framework. Autom Constr. (2013) 34:16–24. doi: 10.1016/j.autcon.2012.10.013

[12] Zhang M Shu L Luo X Yuan M Zheng X. Virtual reality technology in construction safety training: extended technology acceptance model. Autom Constr. (2022) 135:104113. doi: 10.1016/j.autcon.2021.104113

[13] Capasa L Zulauf K Wagner R. Virtual reality experience of mega sports events: a technology acceptance study. J Theor Appl Electron Commer Res. (2022) 17:686–703. doi: 10.3390/jtaer17020036

[14] Liu D Lu W Niu Y. Extended technology-acceptance model to make smart construction systems successful. J Constr Eng Manag. (2018) 144:1–9. doi: 10.1061/(ASCE)CO.1943-7862.0001487

[15] Fishbein M. Ajzen I. Belief, attitude, intention and behaviour: an introduction to theory and research. American Sociological Association (1975).

[16] Ajzen I. The theory of planned behavior. Organ Behav Hum Decis Process. (1991) 50:179–211. doi: 10.1016/0749-5978(91)90020-T

[17] Ajzen I. From intentions to actions: a theory of planned behavior In: Kuhl J Beckmann J, editors. Action control: from cognition to behavior. Berlin, Heidelberg: Springer (1985). 11–39.

[18] Manis KT Choi D. The virtual reality hardware acceptance model (VR-HAM): extending and individuating the technology acceptance model (TAM) for virtual reality hardware. J Bus Res. (2019) 100:503–13. doi: 10.1016/j.jbusres.2018.10.021

[19] Man SS Chan AHS Wong HM. Risk-taking behaviors of Hong Kong construction workers – a thematic study. Saf Sci. (2017) 98:25–36. doi: 10.1016/j.ssci.2017.05.004

[20] Man SS Alabdulkarim S Chan AHS Zhang T. The acceptance of personal protective equipment among Hong Kong construction workers: an integration of technology acceptance model and theory of planned behavior with risk perception and safety climate. J Saf Res. (2021) 79:329–40. doi: 10.1016/j.jsr.2021.09.014, PMID: 34848013

[21] Steuer J. Defining virtual reality: dimensions determining telepresence. J Commun. (1992) 42:73–93. doi: 10.1111/j.1460-2466.1992.tb00812.x

[22] Chih Hung C Jie Chi Y Sarah S Ming Chang J. A desktop virtual reality earth motion system in astronomy education. J Educ Technol Soc. (2007) 10:289–304. doi: 10.2307/2065853

[23] Dubovi I Levy ST Dagan E. Now I know how! The learning process of medication administration among nursing students with non-immersive desktop virtual reality simulation. Comput Educ. (2017) 113:16–27. doi: 10.1016/j.compedu.2017.05.009

[24] Morélot S Garrigou A Dedieu J N'Kaoua B. Virtual reality for fire safety training: influence of immersion and sense of presence on conceptual and procedural acquisition. Comput Educ. (2021) 166:104145. doi: 10.1016/j.compedu.2021.104145

[25] Zhang H He X Mitri H. Fuzzy comprehensive evaluation of virtual reality mine safety training system. Saf Sci. (2019) 120:341–51. doi: 10.1016/j.ssci.2019.07.009

[26] Laycock S Day A. A survey of haptic rendering techniques. Comput Graph Forum. (2007) 26:50–65. doi: 10.1111/j.1467-8659.2007.00945.x

[27] Bowman D Coquillart S Froehlich B Hirose M Kitamura Y Kiyokawa K. 3D user interfaces: new directions and perspectives. IEEE Comput Graph Appl. (2008) 28:20–36. doi: 10.1109/MCG.2008.109, PMID: 19004682

[28] Mitra S Acharya T. Gesture recognition: a survey. IEEE Trans Syst Man Cybernet Part C. (2007) 37:311–24. doi: 10.1109/TSMCC.2007.893280, PMID: 39573497

[29] Davis FD. Perceived usefulness, perceived ease of use, and user acceptance of information technology. MIS Q. (1989) 13:319–40. doi: 10.2307/249008

[30] Lu Y Zhou T Wang B. Exploring Chinese users' acceptance of instant messaging using the theory of planned behavior, the technology acceptance model, and the flow theory. Comput Hum Behav. (2009) 25:29–39. doi: 10.1016/j.chb.2008.06.002, PMID: 39649829

[31] Noor Arzahan IS Ismail Z Yasin SM. Safety culture, safety climate, and safety performance in healthcare facilities: a systematic review. Saf Sci. (2022) 147:105624. doi: 10.1016/j.ssci.2021.105624

[32] Choi YK Totten JW. Self-construal's role in mobile TV acceptance: extension of TAM across cultures. J Bus Res. (2012) 65:1525–33. doi: 10.1016/j.jbusres.2011.02.036

[33] Hu PJ Chau PYK Sheng ORL Tam KY. Examining the technology acceptance model using physician acceptance of telemedicine technology. J Manag Inf Syst. (1999) 16:91–112. doi: 10.1080/07421222.1999.11518247

[34] Ajzen I Fishbein M. Attitude-behavior relations: a theoretical analysis and review of empirical research. Psychol Bull. (1977) 84:888–918. doi: 10.1037/0033-2909.84.5.888

[35] Berger IE Mitchell AA. The effect of advertising on attitude accessibility, attitude confidence, and the attitude-behavior relationship. J Consum Res. (1989) 16:269–79. doi: 10.1086/209213

[36] Hsu CHC Cai LA Mimi L. Expectation, motivation, and attitude: a tourist behavioral model. J Travel Res. (2009) 49:282–96. doi: 10.1177/0047287509349266, PMID: 39624227

[37] Fett D Ensslen A Jochem P Fichtner W. A survey on user acceptance of wireless electric vehicle charging. World Electric Vehicle J. (2018) 9:36. doi: 10.3390/wevj9030036

[38] Lili D Ying Y Qiuhui H Mengxi L. Residents’ acceptance of using desalinated water in China based on the theory of planned behaviour (TPB). Mar Policy. (2021) 123:104293. doi: 10.1016/j.marpol.2020.104293

[39] Liao C Huang Y Zheng Z Xu Y. Investigating the factors influencing urban residents’ low-carbon travel intention: a comprehensive analysis based on the TPB model. Transp Res Interdis Perspect. (2023) 22:100948. doi: 10.1016/j.trip.2023.100948

[40] Han H Kim Y. An investigation of green hotel customers’ decision formation: developing an extended model of the theory of planned behavior. Int J Hosp Manag. (2010) 29:659–68. doi: 10.1016/j.ijhm.2010.01.001

[41] Hsiao C-H Yang C. Predicting the travel intention to take high speed rail among college students. Transport Res F: Traffic Psychol Behav. (2010) 13:277–87. doi: 10.1016/j.trf.2010.04.011

[42] Slovic P. Perception of risk. Science. (1987) 236:280–5. doi: 10.1126/science.3563507, PMID: 3563507

[43] Ooi K-B Tan GW-H. Mobile technology acceptance model: an investigation using mobile users to explore smartphone credit card. Expert Syst Appl. (2016) 59:33–46. doi: 10.1016/j.eswa.2016.04.015

[44] Schierz PG Schilke O Wirtz BW. Understanding consumer acceptance of mobile payment services: an empirical analysis. Electron Commer Res Appl. (2010) 9:209–16. doi: 10.1016/j.elerap.2009.07.005

[45] Yang Y Liu Y Li H Yu B. Understanding perceived risks in mobile payment acceptance. Ind Manag Data Syst. (2015) 115:253–69. doi: 10.1108/IMDS-08-2014-0243

[46] Huijts NMA Molin EJE Steg L. Psychological factors influencing sustainable energy technology acceptance: a review-based comprehensive framework. Renew Sust Energ Rev. (2012) 16:525–31. doi: 10.1016/j.rser.2011.08.018

[47] Man SS Xiong W Chang F Chan AHS. Critical factors influencing acceptance of automated vehicles by Hong Kong drivers. IEEE Access. (2020) 8:109845–56. doi: 10.1109/ACCESS.2020.3001929

[48] Man SS Chan AHS Alabdulkarim S. Quantification of risk perception: development and validation of the construction worker risk perception (CoWoRP) scale. J Saf Res. (2019) 71:25–39. doi: 10.1016/j.jsr.2019.09.009, PMID: 31862036

[49] Seo H-C Lee Y-S Kim J-J Jee N-Y. Analyzing safety behaviors of temporary construction workers using structural equation modeling. Saf Sci. (2015) 77:160–8. doi: 10.1016/j.ssci.2015.03.010

[50] Shin D-P Gwak H-S Lee D-E. Modeling the predictors of safety behavior in construction workers. Int J Occup Saf Ergon. (2015) 21:298–311. doi: 10.1080/10803548.2015.1085164, PMID: 26414618

[51] Barling J Loughlin C Kelloway EK. Development and test of a model linking safety-specific transformational leadership and occupational safety. J Appl Psychol. (2002) 87:488–96. doi: 10.1037/0021-9010.87.3.488, PMID: 12090606

[52] Zohar D Luria G. A multilevel model of safety climate: cross-level relationships between organization and group-level climates. J Appl Psychol. (2005) 90:616–28. doi: 10.1037/0021-9010.90.4.616, PMID: 16060782

[53] Pandit B Albert A Patil Y Al-Bayati AJ. Impact of safety climate on hazard recognition and safety risk perception. Saf Sci. (2019) 113:44–53. doi: 10.1016/j.ssci.2018.11.020

[54] Fugas CS Silva SA Meliá JL. Another look at safety climate and safety behavior: deepening the cognitive and social mediator mechanisms. Accid Anal Prev. (2012) 45:468–77. doi: 10.1016/j.aap.2011.08.013, PMID: 22269531

[55] Bowman DA McMahan RP. Virtual reality: how much immersion is enough? Computer. (2007) 40:36–43. doi: 10.1109/MC.2007.257

[56] Cobb SVG Nichols S Ramsey A Wilson JR. Virtual reality-induced symptoms and effects (VRISE). Presence Teleop Virt. (1999) 8:169–86. doi: 10.1162/105474699566152

[57] Kim HK Park J Choi Y Choe M. Virtual reality sickness questionnaire (VRSQ): motion sickness measurement index in a virtual reality environment. Appl Ergon. (2018) 69:66–73. doi: 10.1016/j.apergo.2017.12.016, PMID: 29477332

[58] Yi MY Hwang Y. Predicting the use of web-based information systems: self-efficacy, enjoyment, learning goal orientation, and the technology acceptance model. Int J Hum Comput Stud. (2003) 59:431–49. doi: 10.1016/S1071-5819(03)00114-9

[59] Fornell C Larcker D. Evaluating structural equation models with unobservable variables and measurement error. J Mark Res. (1981) 18:39–346. doi: 10.2307/3151312, PMID: 38532229

[60] Kang S Kim I Lee K. Predicting deviant behaviors in sports using the extended theory of planned behavior. Front Psychol. (2021) 12:678948. doi: 10.3389/fpsyg.2021.678948, PMID: 34566759 PMC8459903

[61] Zhao J Wijaya TT Mailizar M Habibi A. Factors influencing student satisfaction toward STEM education: exploratory study using structural equation modeling. Appl Sci. (2022) 12:9717. doi: 10.3390/app12199717

[62] Sim J-J Tan GW-H Wong JCJ Ooi K-B Hew T-S. Understanding and predicting the motivators of mobile music acceptance – a multi-stage MRA-artificial neural network approach. Telematics Inform. (2014) 31:569–84. doi: 10.1016/j.tele.2013.11.005

[63] Cronbach LJ. Coefficient alpha and the internal structure of tests. Psychometrika. (1951) 16:297–334. doi: 10.1007/BF02310555

[64] Ab Hamid MR Sami W Mohmad Sidek MH. Discriminant validity assessment: use of Fornell & amp; Larcker criterion versus HTMT criterion. J Phys Conf Ser. (2017) 890:012163. doi: 10.1088/1742-6596/890/1/012163

[65] Kalinić Z Marinković V Kalinić L Liébana-Cabanillas F. Neural network modeling of consumer satisfaction in mobile commerce: an empirical analysis. Expert Syst Appl. (2021) 175:114803. doi: 10.1016/j.eswa.2021.114803

[66] Ahani A Rahim NZA Nilashi M. Forecasting social CRM adoption in SMEs: a combined SEM-neural network method. Comput Hum Behav. (2017) 75:560–78. doi: 10.1016/j.chb.2017.05.032

[67] Eiris R Gheisari M Esmaeili B. Desktop-based safety training using 360-degree panorama and static virtual reality techniques: a comparative experimental study. Autom Constr. (2020) 109:102969. doi: 10.1016/j.autcon.2019.102969

[68] Goulding J Nadim W Petridis P Alshawi M. Construction industry offsite production: a virtual reality interactive training environment prototype. Adv Eng Inform. (2012) 26:103–16. doi: 10.1016/j.aei.2011.09.004

[69] Shi C Miao X Liu H Han Y Wang Y Gao W. How to promote the sustainable development of virtual reality technology for training in construction filed: a tripartite evolutionary game analysis. PLoS One. (2023) 18:e0290957. doi: 10.1371/journal.pone.0290957, PMID: 37656741 PMC10473506

[70] Rey-Becerra E Barrero LH Ellegast R Kluge A. Improvement of short-term outcomes with VR-based safety training for work at heights. Appl Ergon. (2023) 112:104077. doi: 10.1016/j.apergo.2023.104077, PMID: 37369152

[71] Hu X Griffin MA Bertuleit M. Modelling antecedents of safety compliance: incorporating theory from the technological acceptance model. Saf Sci. (2016) 87:292–8. doi: 10.1016/j.ssci.2015.12.018

[72] Najmi A Kanapathy K Aziz AA. Understanding consumer participation in managing ICT waste: findings from two-staged structural equation modeling–artificial neural network approach. Environ Sci Pollut Res. (2021) 28:14782–96. doi: 10.1007/s11356-020-11675-2, PMID: 33219501

[73] Nykänen M Puro V Tiikkaja M Kannisto H Lantto E Simpura F. Implementing and evaluating novel safety training methods for construction sector workers: results of a randomized controlled trial. J Saf Res. (2020) 75:205–21. doi: 10.1016/j.jsr.2020.09.015, PMID: 33334479

[74] Dinis FM Guimarães AS Carvalho BR Martins JPP. Development of virtual reality game-based interfaces for civil engineering education, 2017 IEEE global engineering education conference (EDUCON), pp. 1195–1202. (2017).

[75] Guo H Li H Chan G Skitmore M. Using game technologies to improve the safety of construction plant operations. Accid Anal Prev. (2012) 48:204–13. doi: 10.1016/j.aap.2011.06.002, PMID: 22664683

[76] Lee J Kim J Choi JY. The adoption of virtual reality devices: the technology acceptance model integrating enjoyment, social interaction, and strength of the social ties. Telematics Inform. (2019) 39:37–48. doi: 10.1016/j.tele.2018.12.006

[77] Lin P-H Yeh S-C. How motion-control influences a VR-supported technology for mental rotation learning: from the perspectives of playfulness, gender difference and technology acceptance model. Int J Hum Comput Interact. (2019) 35:1736–46. doi: 10.1080/10447318.2019.1571784

[78] Padilla-Meléndez A del Aguila-Obra AR Garrido-Moreno A. Perceived playfulness, gender differences and technology acceptance model in a blended learning scenario. Comput Educ. (2013) 63:306–17. doi: 10.1016/j.compedu.2012.12.014

[79] Bhavani B. Sheshadri S. Radhakrishnan U. Vocational education technology: rural India. India: Amrita ACM-W Celebration on Women in Computing. (2010).

[80] Schuemie MJ Peter VDS Krijn M van der Mast CAPG. Research on presence in virtual reality: a survey. Cyber Psychol Behav. (2001) 4:183–201. doi: 10.1089/109493101300117884, PMID: 11710246

[81] Davis FD Warshaw BPR. User acceptance of computer-technology - a comparison of 2 theoretical-models. Manag Sci. (1989) 35:982–1003. doi: 10.1287/mnsc.35.8.982, PMID: 19642375

[82] Venkatesh V. Determinants of perceived ease of use: integrating perceived behavioral control, computer anxiety and enjoyment into the technology acceptance model. Inf Syst Res. 11:342–65.

[83] Chang C-T Hung M-F. Mind the gap: analyzing factors associated with consumers' single-use product reduction. Sustain Prod Consum. (2023) 36:75–87. doi: 10.1016/j.spc.2022.12.019

[84] Wang B Li Y. Consumers’ intention to bring a reusable bag for shopping in China: extending the theory of planned behavior. Int J Environ Res Public Health. (2022) 19:3638. doi: 10.3390/ijerph19063638, PMID: 35329325 PMC8955543

